# Case Report: Whole Exome Sequencing Revealed Two Novel Mutations of *PIEZO1* Implicated in Nonimmune Hydrops Fetalis

**DOI:** 10.3389/fgene.2021.684555

**Published:** 2021-08-05

**Authors:** Yuan Chen, Ying Jiang, Bangwu Chen, Yeqing Qian, Jiao Liu, Mengmeng Yang, Baihui Zhao, Qiong Luo

**Affiliations:** ^1^Department of Obstetrics, Women's Hospital, Zhejiang University School of Medicine, Hangzhou, China; ^2^Department of Obstetrics, Ninghai Maternal and Child health Care Hospital, Ningbo, China; ^3^Department of Reproductive Genetics, Women's Hospital Zhejiang University School of Medicine, Hangzhou, China; ^4^Department of Obstetrics, Lishui Maternal and Child Health Care Hospital, Lishui, China

**Keywords:** whole exome sequencing, nonimmune hydrops fetalis, PIEZO1 gene, sanger sequencing, case report

## Abstract

Nonimmune hydrops fetalis (NIHF) is a serious and complex fetal condition. Prenatal diagnosis of hydrops fetalis is not difficult by ultrasound. However, determining the underlying etiology of NIHF remains a challenge which is essential to address for prenatal counseling. We extracted DNA from a proband prenatally diagnosed unexplained NIHF. Trio-whole exome sequencing (WES) was performed to filter candidate causative variants. Two gene mutations were identified as a compound heterozygous state in the proband. Both variants located on the *PIEZO1* gene: c.3895C > T, a missense mutation in exon 27 paternally inherited; c.4030_4032del, a maternally inherited in-frame deletion in exon 28. Both variants were first reported to be related to NIHF. *PIEZO1* gene mutations, leading to an autosomal recessive congenital lymphatic dysplasia, which can present as NIHF and partial or complete resolution postnatally. In conclusion, WES can aid in the elucidation of the genetic cause of NIHF and has a positive effect on the assessment of prognosis.

## Introduction

Nonimmune hydrops fetalis (NIHF) is a rare and serious congenital disease, accounting for about 90% of hydrops fetalis cases, with a prevalence estimated to be ~0.03%~0.06% (Norton et al., 2015). The underlying pathogenesis of NIHF is complicated, ranging from cardiovascular, chromosomal, hematologic, infectious, thoracic, twin-twin transfusion, urinary tract abnormalities, gastrointestinal, lymphatic dysplasia, tumors, skeletal dysplasias, syndromic, inborn errors of metabolism, miscellaneous, and other unknown causes. The prognosis of NIHF appears poor generally (mortality reported is over 50%) (Norton et al., 2015). Despite the rapid development of intrauterine therapy, the decisions and survival rates of prenatal management depend on the definable and treatable causes. Therefore, identifying the underlying cause of NIHF is particularly important for prenatal counseling. The Society of Maternal-Fetal Medicine (SMFM) published clinical guidelines for NIHF in 2015. Targeted ultrasonic evaluation, middle cerebral artery (MCA) Doppler, fetal karyotype or chromosomal microarray analysis, and polymerase chain reaction (PCR) for cytomegalovirus, parvovirus, toxoplasmosis are recommended as the first-line workup of NIHF (Norton et al., 2015). Even with standard evaluations, the cause remains unknown in 20% to 68% of cases as reported (Sparks et al., 2019).

In recent years, genetic testing has been widely used to identify the causes of fetal anomalies. Traditional chromosome karyotyping by G-banding technique accounted for 9%~19% of fetal structural abnormalities. Chromosomal microarray analysis (CMA) further reveals additional findings in 6% of cases (Staebler et al., 2005; Wapner et al., 2012; Norton et al., 2015; Levy and Wapner, 2018). In a large and multicenter cohort study (65 NIHF cases were included), the underlying etiology of prenatally diagnosed NIHF was determined in 44% of cases, and a genetic cause was identified in 25% of those receiving standard genetic testing (Sparks et al., 2019). More recently, WES has been applied to explain those cases with normal results of karyotype and CMA. In a prospective cohort study enrolling 234 parents-fetus trios with structural anomalies but were negative in karyotype and chromosomal microarray findings. The genetic diagnostic yield of WES was approximately 10% (Petrovski et al., 2019). In another large case series of unexplained NIHF (127 cases were included), diagnostic genetic variants were identified in 29% of cases by WES (Sparks et al., 2020). A total of 131 genes were summarized with strong evidence for a relationship with NIHF and further categorized as follows: cardiovascular, inborn metabolic issues, hematological, lymphatic, skeletal, neuromuscular, syndromic disorders. Lymphatic disorders play an important part in the etiology of NIHF and comprise 15% of NIHF (Bellini et al., 2015). Based on strong and emerging evidence genes, the genetic variants associated with lymphatic malformations account for approximately 5.1% of NIHF, including *FLT4, CCBE1, ADAMITS3, PIEZO1, EPHB4, SOX18*, and *FOXC2* (Quinn et al., 2021).

*PIEZO1* is known to encode a mechanically activated ion channel in the plasma membrane of several types of cells (including hepatic erythroblasts, fetal splenic plasma cells, and lymphatic vessels of fetal peritoneum) and highly conserved in mammalia (Coste et al., 2010; Andolfo et al., 2013). *PIEZO1* gain of function mutations is identified to be the cause of autosomal dominant dehydrated hereditary stomatocytosis (DHS) (OMIM 194380), which is a rare genetic cause of hemolytic anemia. Biallelic *PIEZO1* loss-of-function mutations were first reported in 2015 to relate with other different phenotypes. These include autosomal recessive congenital lymphatic dysplasia (GLD) (OMIM 616843), which is one rare cause of NIHF *in utero* and can present as generalized lymphoedema involving hydrothorax, hydropericardium, chylothorax, facial and extremities lymphoedema, intestinal or pulmonary lymphangiectasia (Fotiou et al., 2015; Lukacs et al., 2015).

More and more genetic defects are revealed to have a relationship with NIHF (Norton et al., 2015; Sparks et al., 2020). Some cases may be accompanied by characteristic manifestations which can be screened by prenatal sonographic scannings. Yet this is not always the case, such as in lymphatic disorders. In those circumstances, WES is considered a potential technique to aid in the systematic evaluation of unexplained NIHF cases (Sparks et al., 2020; Quinn et al., 2021).

In our study, we performed whole exome sequencing in a case of prenatally diagnosed NIHF. Two novel homozygous variants in *PIEZO1* were identified which were suspected to be the pathogenesis of lymphatic dysplasia with NIHF.

## Materials and Methods

### Patient Information

**Figure 1:**
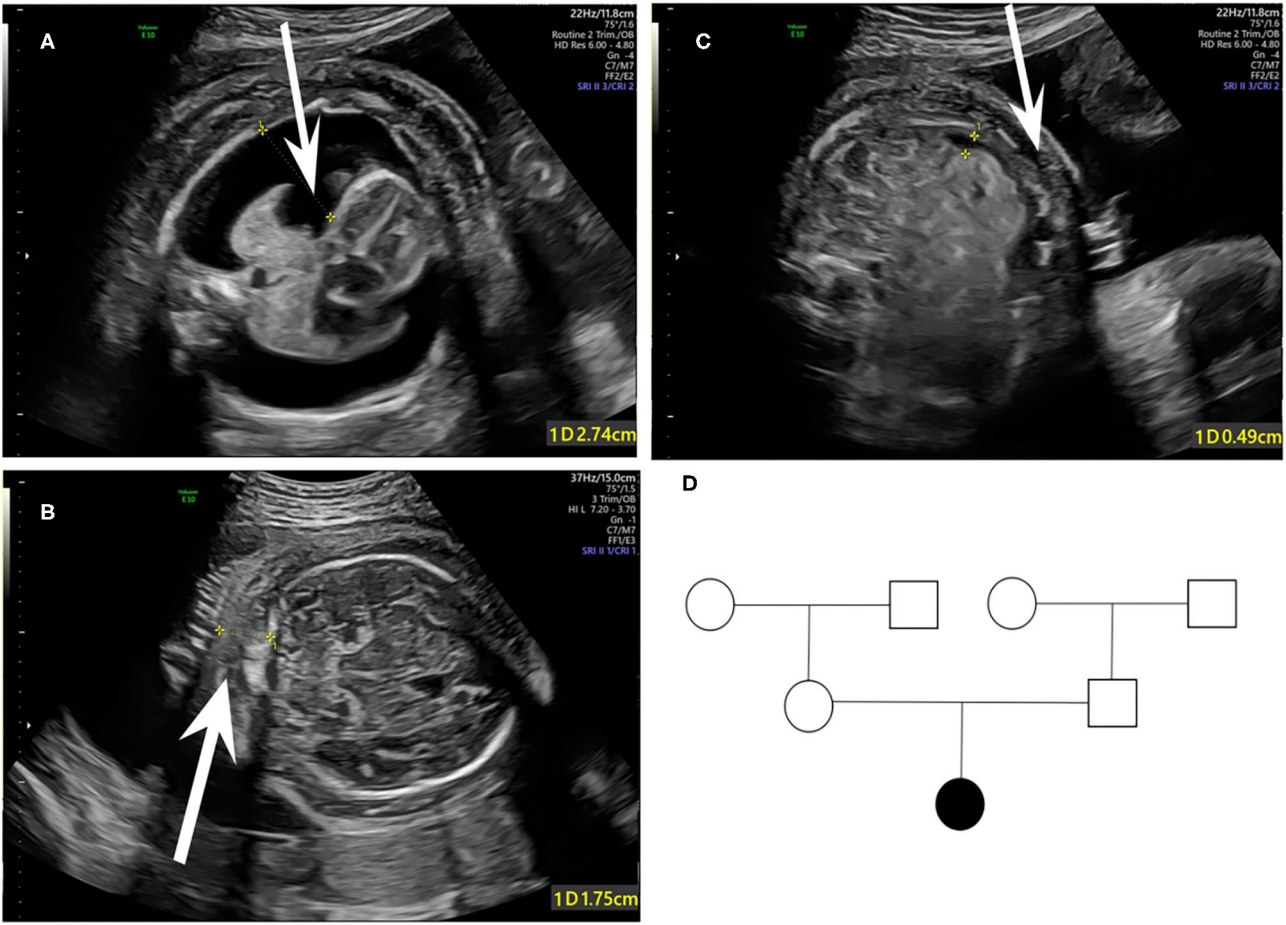
Fetal sonographic findings at 31 gestation in the NIHF case with *PIEZO1* mutations: **(A)** Bilateral pleural effusion on axial view. **(B)** Skin edema on axial view. **(C)** Peritoneal effusion on axial view. **(D)** Pedigree of the family. The affected fetus is marked in black.

A 33-year-old primipara presented at 30 weeks gestation with fetal hydrothorax examined by routine prenatal ultrasound. These parents were referred to our prenatal diagnosis center for further confirmation. No remarkable abnormalities of prior prenatal examinations during pregnancy were found, including normal nuchal fold thickness and low risk prenatal serum screening results (chromosomes 18, 21, and NTD). Both of the parents denied a family history of hydrops fetalis, congenital metabolic diseases, and lymphoedema. At 31 weeks gestation, a thorough fetal systematic ultrasound was performed by prenatal diagnostic ultrasound specialists and showed massive bilateral pleural effusion, generalized skin edema (skin thickness of head and neck region was 18 mm), minor ascites, and polyhydramnios (amniotic fluid index was 28.5 cm) (Figures 1A–C). No other structural anomalies were found. Then systemic evaluations about hydrops fetalis showed Rh-positive blood type, negative serologic testing for TORCH, normal blood urine routine examinations, hepatorenal function, and fetal echocardiogram. After comprehensive counseling, the couples declined *utero* fetal therapy including fetal thoracentesis or placement of thoracoamniotic shunt for drainage even though the underlying etiology of NIHF was unclear. For fear of poor prognosis, the parents terminated the pregnancy at 31 weeks. A female fetus was delivered with a birth weight of 2,610 g and presented as anasarca in appearance. Further autopsy and pathology information for the fetus was not available.

### Ethics Approval

After giving full authorization and signing a written informed consent by both of the parents, whole exome sequencing (WES) (trio analysis of the proband, mother, and father) was carried out. Our study was approved by the Ethics Committee of the Women's Hospital, Zhejiang University, School of Medicine (approval number: IRB-20210124-R) and conformed with ethical standards of experiments on human subjects.

### DNA Extraction

For DNA sampling, 5 ml peripheral whole blood was collected from the biological parents, and a medial thigh muscle tissue (1 × 1 × 1 cubic centimeter) was obtained from the aborted fetus. Then EDTA was added for anticoagulation. A Qiagen Blood DNA mini kit (Qiagen®, Germany) was applied to extract genomic DNA from 2 ml peripheral whole blood of the parents. In addition, a Genomic DNA Purification Kit (*Invitrogen*, cat. K0512, USA) was used for DNA extraction from the muscle sample. Then all of the DNA samples were preserved at −20°C. The remaining samples were stored at −80°C refrigeration.

### Whole Exome Sequencing and Data Analysis

The detailed exome sequencing process for the proband sample and data filtering protocol have been described previously (Jiang et al., 2019). In brief, for targeted enrichment of target region sequences, the precapture library was enriched by multiple probe hybridization using Agilent SureSelect Human Exon Sequence Capture Kit (Agilent, USA). The target region included the coding sequence of the target gene and its neighboring 10 bp intron region. The quality control testing of the sequencing data was achieved by Qubit (Agilent High Sensitivity DNA Kit, Agilent). The postcapture sequencing libraries were analyzed by Illumina DNA Standa ds and Primer Premix Kit (Kapa Biosystems, USA). All filtered variants by new-generation sequencing (NGS) using Illumina HiSeq 2,500 platform (Illumina, USA) were further validated by Sanger sequencing in the proband and parental samples. Fastp (v.0.12.0) was used for quality control of fastq files by using the -N 15-L 30 parameters and low quality reads were filtered out from all sequencing dates using a quality score of 20 (Q20).

The sequencing data were compared to the GRCh37/hG19 reference sequence of the human genome using BWA (v.0.7.17-r1188) with MEM align method. Then variants were called by GATK (v.4.0) and annotated to public databases by Annovar. Pre-treatment for variant calling included: 1) sorting the matched SAM file according to the chromosomal position, and converting it into the compressed binary file BAM by Picard-SortsAM. 2) Adding a sample ID for matched reads by Picard-Add Or Replace Read Groups. 3) Marking the same sequence created by PCR duplication, optical duplication by Picard-Mark Duplicates. 4) GATK-Baserecalibrator and GATK-ApplyBQSR were used for site quality correction according to the known SNP and InDel sites of 1,000 Genomes Project. 5) GATK-Haplotypecaller was used for variant identification and forming a VCF file. Susceptibility genes, mitochondrial genes, genes related to polygenetic diseases, and complex diseases were not included in this analysis.

### Bioinformatics Analysis of Variants

The comparison for all filtered single nucleotide variants (SNVs) and small insertions/deletions with the population were conducted on several public databases, including Human Gene Mutation Database (HGMD) (http://www.hgmd.org), Clinvar (http://www.ncbi.nlm.nih.gov/clinvar), Exome Aggregation Consortium (ExAC) (http://exac.broadinstitute.org), Genome Aggregation Database (gnomAD), 1,000 Genomes (http://exac.broadinstitute.org) database, and Genome Asia 1,000k (http://www.genomeasia100k.com/) database, to exclude the mutation sites as known polymorphic sites. The genetic effects and evolutionary conservation of novel variants were further predicted and estimated using SIFT (http://sift.jcvi.org), PROVEAN, Polyphen2 (http://genetics.bwh.harvard.edu/pph2) and Mutation Taster (http://www.mutationtaster.org) programs. The domain information of the protein was retrieved from UniProt (https://www.uniprot.org/). The pathogenicity of identified variants was interpreted according to the American College of Medical Genetics and Genomics (ACMG) standards and guidelines (Richards et al., 2015). Multiple sequence alignment was conducted using NCBI HomoloGene and DNAMAN program to compare *PIEZO1* protein sequences with different species including *H. sapiens, P. troglodytes, M. mulatta, C. lupus, B. taurus, M. musculus*, and *R. norvegicus*. The variants interpretation and ACMG classification are based on the current understanding of the disease and variants at the time of documenting.

## Results

Coverage information for WES is provided in Supplementary Table 1. The trio-based WES detected two novel heterozygous mutations in the *PIEZO1* gene (NM_001142864.3) of the proband: a missense variant (exon 27, Chr16:88792765: c.3895C>T) and an in-frame deletion (exon 28, Chr16:88792029-88792031: c.4030_4032del) (Table 1). The family pedigree is presented in Figure 1D.

**Table 1:** Diagnostic variants of the proband and parents.

**Sample**	**Gene**	**Exome**	**Chromosome**	**HGVS DNA**	**HGVS Protein**	**Heterozygosity**	**Inheritance**	**Classification**
Proband	*PIEZO1*	Exon27	Chr16:88792765	c.3895C > T	p.R1299C	Heterozygous	paternal	VUS
	*PIEZO1*	exon28	Chr16:88792029-88792031	c.4030_4032del	p.E1344del	Heterozygous	Maternal	VUS
Father	*PIEZO1*	exon27	Chr16:88792765	c.3895C > T	p.R1299C	Heterozygous	–	VUS
	*PIEZO1*	exon28	Chr16:88792029-88792031	c.4030_4032del	p.E1344del	Not detected	–	–
Mather	*PIEZO1*	exon27	Chr16:88792765	c.3895C > T	p.R1299C	Not detected	–	–
	*PIEZO1*	exon28	Chr16:88792029-88792031	c.4030_4032del	p.E1344del	Heterozygous	–	VUS

*HGVS, human genome variation society; VUS, variants of uncertain significance*.

**Figure 2:**
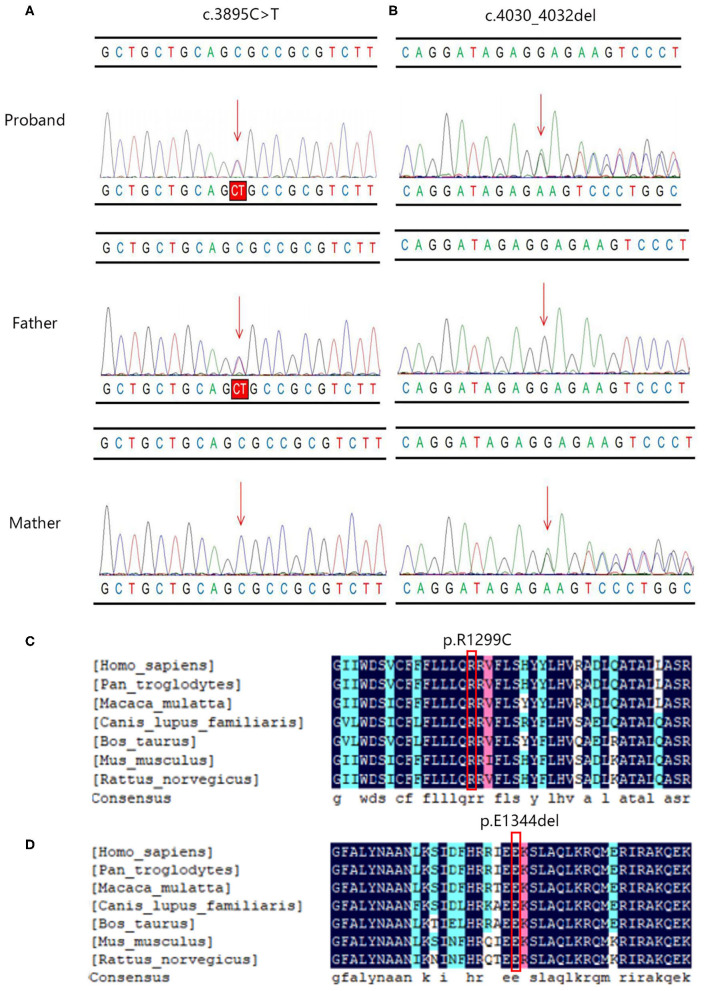
The Sanger sequencing and conservation analysis of *PIEZO1* variants. The chromatograms demonstrated the compound heterozygous status of **(A)** c.3895C > T and **(B)** c.4030_4032del in the *PIEZO1* gene in the proband, proband's father, and proband's mother. The position of the variant or the corresponding wild type nucleotide are labeled with red arrows. Conservation analysis shows that the affected amino acids at positions 1,229 **(C)** and 1,344 **(D)** are located in a highly conserved region across species.

The c.3895C > T variant generated a single amino acid substitution from Arg to Cys at position 1,299 (p.R1299C) in exon 27 of the *PIEZO1* gene located at chromosome 16, which was paternally inherited (Figure 2A). To date, the variant c.3895C > T is not present in HGMD, Clinvar, ExAC, gnomAD, or the 1000 Genomes database (PM2). Bioinformatics analysis predicted the novel variant (NM_001142864.3: c.3895C > T, p.R1299C) to be deleterious by SIFT (score 0.000) and PROVEAN (score −6.84). Mutation Taster suggested that this mutation was located in a highly evolutionarily conserved site, leading to a change of amino acid sequence. Protein features might also be affected (PP3).

The c.4030_4032del variant detected from the proband was located in exon 28 of the *PIEZO1* gene, resulting in an in-frame deletion of glutamic acid at position 1,344 (p. E1344del) and a change in protein length (PM4). According to the results by Sanger sequencing, we found that the in-frame deletion variant was maternally inherited and the unaffected mother was heterozygous for this mutation (Figure 2B). c.4030_4032del have not been reported in the HGMD and Clinvar databases, although the gnomAD database reported an extremely minor population frequency of the mutation as 0.00002568 (PM2). However, no homozygote has been reported, and no carriers of the mutation have been found in East Asian populations. According to the information obtained from the Uniprot website, the amino acid at position 1,344 is located on a coiled coil domain (1,339–1,368) of the PIEZO1 ion channel pore. The coiled coil is a highly versatile folding motif that may determine many biological functions involving signal transduction, molecular recognition, protein refolding, and ion channel formation (Burkhard et al., 2001). One amino acid deletion was supposed to affect the stability or rigidity of the coiled coils motif. p.Ile1357Val, p.Ile1357Met, p.R1358P, and p.Q1361R, which are adjacent to position 1,344, have been previously described as pathogenic mutations leading to autosomal dominant dehydrated stomatocytosis (Albuisson et al., 2013; Kager et al., 2018; Picard et al., 2019). The two mutations associated with amino acids positions were both located in conserved functional domains, which were deeply conserved across species (Figures 2C,D).

## Discussion

By whole exome sequencing, we identified two heterozygous *de novo* variants of *PIEZO1* in an unexplained NIHF case by routine ultrasound examinations prenatally. One was a novel missense mutation in exon 27, inherited from the father of proband. The other was a maternally inherited in-frame deletion in exon 28. Both variants were firstly reported in the PIEZO1 gene, and PIEZO1-associated autosomal recessive congenital lymphatic dysplasia is one rare cause of NIHF in *utero*.

Hydrops fetalis is defined as the presence of two or more abnormal fluid collections in the fetus by prenatal ultrasonography. The sonographic features include ascites, pleural effusions, pericardial effusions, and generalized skin edema (defined as skin thickness over 5 mm) (Norton et al., 2015). Hydrops fetalis is usually considered a lethal fetal condition and the affected mother may suffer from mirror syndrome (Désilets et al., 2018). According to the pathophysiology of hydrops fetalis, the etiology is classified as immune and nonimmune (Norton et al., 2015). With the routine examination of maternal Rh(D) type and application of preventive Rh(D) immunoglobulin, the incidence rate of red cell alloimmunization associated with hydrops fetalis is decreased. Combining thorough ultrasound scanning with fetal MRI, the majority of structural abnormalities associated with NIHF can be prenatally diagnosed (Norton et al., 2015). Even though, it is still a challenging task to elucidate the exact etiology of NIHF, which is essential for prenatal counseling and determination of the appropriate therapy if available (Norton et al., 2015). In recent years, whole exome sequencing (WES) has been gradually applied to identify *de-novo* mutations for evaluation of fetal abnormalities, which is insufficient by conventional molecular approaches such as karyotyping and chromosomal microarray analysis (CMA), including NIHF (Sparks et al., 2020; Quinn et al., 2021).

In our study, the affected proband is compound heterozygotes for a missense variant and a single amino acid in-frame deletion in the *PIEZO1* gene, both of which were not previously reported. Up to now, approximately 13 mutations in the *PIEZO1* gene have been reported to present as GLD associated with NIHF, which consist of six missense variants, four nonsense mutations, and three splicing mutations (Yates et al., 2017; More et al., 2020). The effects of *PIEZO1* variants present significantly more severe prenatally, while the complete or partial resolution of the edema postnatally has been reported in some patients. Fotiou et al. identified 10 *PIEZO1* mutations in six families which cosegregate with the GLD phenotype. The affected individuals suffer from *in utero* demise, complete resolution after birth, the reappearance of lymphoedema in peripheries at early childhood, or recurrent facial cellulitis (Quinn et al., 2021).

Along with development, the junctions of lymphatic endothelial cells transform from zippers into buttons and buttons initially appear at birth, which means a significant change occurs in lymphatics during prenatal and postnatal development. In addition, dexamethasone has promoted button formation during the early development period after birth (Yates et al., 2017). Another study analyzed *PIEZO1* protein expression in human fetal tissues. An obvious positive result was observed in fetal lymphatic vasculature while the immunoreactivity of *PIEZO1* protein was completely negative in healthy adult subjects (Andolfo et al., 2013). Based on these findings *in vitro*, one reasonable inference is that *PIEZO1* plays an important role in maintaining the lymphatic system *in utero* state, but has a decreased significance at neonate state. The clinical outcomes of *PIEZO1* associated NIHF range from fetal demise to a complete resolution after birth (Martin-Almedina et al., 2018). Whether various clinical outcomes are related to different mutations of the *PIEZO1* gene remains unclear. Questions remain regarding the kinds of variants in *PIEZO1* that result in poor outcomes or which can even be lethal, and which kind of variants have a good prognosis and are worthy of aggressive treatment.

In our report, the clinical manifestation of proband was severe hydrops fetalis without accompanied fetal structural abnormalities. This was determined by searching population frequency databases and undertaking bioinformatics analysis using several software packages, c.3895C > T and c.4030_4032del mutations are not suspected to be benign variations. Due to the lack of an identified human PIEZO1 channel structure by experimental methods, high-resolution cryo-electron microscopy (cryo-EM) structure and the mechanogating mechanism of the mouse PIEZO1 channel have been determined (Zhao et al., 2018). The residues H1300 to S1362 are assigned to the beam structure, which is considered to act as a lever-like mechanotransduction apparatus for converting the tension-induced conformational change of the peripheral blade to the intracellular gate. The two mutations are located beside and in the key structure and are supposed to affect the conductance and mechanosensitivity of the PIEZO1 channel. However, the exact biological effect needs to be further verified according to more functional experiments. In addition, when combined with the carrier state of the parents, as confirmed by Sanger sequencing, the inherited pattern is consistent with the *PIEZO1* associated autosomal recessive congenital lymphatic dysplasia. Thus, the phenotype of the patient could be considered as supporting evidence (PP4). Since well-established functional studies *in vivo* or *in vitro* of a damaging effect on the mutations were not available and reported information was rare, both variants were classified as a variant of unknown clinical significance (VUS) based on ACMG guidelines (c.3895C > T: PM2 + PP3 + PP4; c.4030_4032del: PM2+PM4+PP4).

In conclusion, our report suggested that two novel variants (c.3895C > T and c.4030_4032del) identified in *PIEZO1* could be associated with NIHF, which might be a severe phenotype of GLD *in utero*. These results provide additional information that reveals the specific mechanisms underlying PIEZO1-related lymphatic development and function. Our data also advocates the application of WES to further detect the underlying genetic cause of unexplained NIHF by standard genetic testing, which is important for making an accurate diagnosis for the precise evaluation of the recurrence risk and prognosis, detailed prenatal counseling, and high-quality perinatal and postnatal management.

## Data Availability Statement

The data that support the findings of this study are available on request.

## Ethics Statement

The studies involving human participants were reviewed and approved by Ethics Committee of the Women's Hospital, Zhejiang University, School of Medicine. The patients/participants provided their written informed consent to participate in this study. Written informed consent was obtained from the individual(s) for the publication of any potentially identifiable images or data included in this article.

## Author Contributions

YC, YJ, and QL designed the study and wrote the manuscript. MY and BC undertook patients care, collecting samples and clinical information. YC, YQ, and BZ undertook bioinformatics analysis. JL and QL revised the manuscript. All authors reviewed the manuscript and approved the final version.

## Conflict of Interest

The authors declare that the research was conducted in the absence of any commercial or financial relationships that could be construed as a potential conflict of interest.

## Publisher's Note

All claims expressed in this article are solely those of the authors and do not necessarily represent those of their affiliated organizations, or those of the publisher, the editors and the reviewers. Any product that may be evaluated in this article, or claim that may be made by its manufacturer, is not guaranteed or endorsed by the publisher.
