# Supplementary material for: Case Report: Whole Exome Sequencing Revealed Two Novel Mutations of PIEZO1 Implicated in Nonimmune Hydrops Fetalis
Source: Front Genet. 2021 Aug 5;12:684555. doi: 10.3389/fgene.2021.684555 (PMC8375471; doi:10.3389/fgene.2021.684555)
Supplement: Supplementary file 1 [file Table_1.DOCX]

Supplementary Table 1. Sequencing quality statistics of NGS on the targeted region

| Sample | Number of targeted genes | Coverage of target region (%) | Average sequencing depth on target（X） | Fraction of target covered with at least 20x (%) |
| --- | --- | --- | --- | --- |
| Proband | 22,000 | 99.59 | 107.76 | 96.54 |
| Father | 22,000 | 99.91 | 118.55 | 97.67 |
| Mother | 22,000 | 99.62 | 106.11 | 97.67 |
